# Value-based leadership in turbulent times: lessons from the Corona crisis and recommendations for post-pandemic management in the health sector

**DOI:** 10.1365/s42681-022-00029-w

**Published:** 2022-01-19

**Authors:** Maximilian C. von Eiff, Wilfried von Eiff, Mohamed Ghanem

**Affiliations:** 1 Clinic for Urology, Paediatric Urology und Uro-Gynokology, St. Josef Hospital Hamm (Franziskus Foundation Hospital Group Muenster), Hamm-Bockum-Hövel, Germany; 2 Center for Hospital Management (University of Muenster), Kerckhoff Clinic (Bad Nauheim), Münster, Germany; 3 Department of Orthopedics, Traumatology and Plastic Surgery, Department of Rehabilitation, University Clinic of Leipzig Germany, Leipzig, Germany

**Keywords:** Purpose, Economies of scale, Digitalization, Economization of medicine, Supply shortages, Working conditions “at the bedside”, Corona crisis

## Abstract

The Corona crisis not only exposed the causes of supply disruptions for system-critical medical products and pharmaceuticals, and made the consequences of the digitalization gap in the health care system transparent, but in particular, revealed the consequences of fundamental leadership deficits in hospital personnel management, professional profiles and ethics, professional policies, and procurement management. However, Corona has also triggered a rethinking of the values, meaning and purpose of work content and behavioral norms. This paper aims to identify and analyse management failures observed and experiences made during the Corona pandemic. Based on these findings recommendations for good leadership practices are given. By literature research reported experiences from physicians and nurses were analysed related to working conditions, motivation-to-work, and satisfaction with incentive systems. Furthermore, interviews with clinical staff working under Corona conditions were realised based on a structured questionnaire. The workload of nursing has increased significantly due to economization: from 2005 to 2017, the number of treatment cases increased by 12%, while at the same time, the number of beds decreased by 9.4% and the length of stay shortened from 8.4 to 7.3 days. The accumulated nursing overtime in German hospitals alone is equivalent to 17,800 full-time employees. During the Corona crisis the working situation especially for nurses facilitating patients ventilated on the intensive care unit has dramatically worsened: additional overtime, high patient mortality, resource-intensive and stressful care requirements lead to prostration and mental exhaustion. As a consequence of this tremendous work burden for nurses and physicians during the Corona pandemic up to 30% of these occupational groups gave voice to inadequate working conditions and utter their intention to quit their jobs. Demotivation and a flight into professions remote from medicine are a reaction of many physicians and nurses to years of leadership failures in hospitals and politics, as well as an increasing economization of medicine. Between 68 and 82% of physicians cite the cost pressure associated with rationing as a source of dissatisfaction with their professional situation. It is up to management to learn from these findings, implement the necessary measures and provide family-friendly working conditions for occupational groups working “at the bed-side”. A “value-based leadership model” that takes into account the specific conditions prevailing in the healthcare industry was developed and serves as a compass in meeting and overcoming this challenge. This paper transfers practical experiences made during the Corona pandemic and pertaining to motivation-to-work under stressful working conditions, the meaning of “purpose”, the try-out of so far unknown working practices and types of inter-occupational co-operation into a leadership model that is unique for the health care sector.

## Finding a sense of purpose in work: the importance of “purpose” and “perceived value” in a medical working environment

Finding a sense of purpose in the work, identification with the profession, and awareness of contributing to the common good as part of the provision of public welfare should not be problems for medical staff in principle. Medical ethics and nursing ethics focus on helping sick people at the existential, physical and psychological extreme, in borderline situations. This is about people whose behavior is characterized by fear, uncertainty, pain and a loss of autonomy. Taking responsibility in this context, and demonstrating entrepreneurial awareness in the sense of patient-centeredness, continuous quality improvement and a willingness to solve problems, is the normal work routine for physicians and nurses, and is what keeps the medical sector operating at a high level of efficiency. Supporting professions such as medical assistants, medical controllers and purchasers also draw motivation and identification from being part of a medical performance process that is significant for both society and the economy.

Furthermore, attractive working conditions are a convincing instrument of human resource management in order to achieve staff retention and successful recruitment (von Eiff 2019a, b). On one hand working conditions from the employees point of view are perceived as an expression of appreciation for the importance of an occupational group. On the other hand working conditions as well as leadership style contribute to staff satisfaction and determine the willingness to a commitment for carrying out patient-centered services. At the end of the day it is fair to say that patient satisfaction, patient readiness for recommendation and achieving a brand or magnet status are the consequence of motivating working conditions (see appendix Fig. 1).

**Fig. 1:**
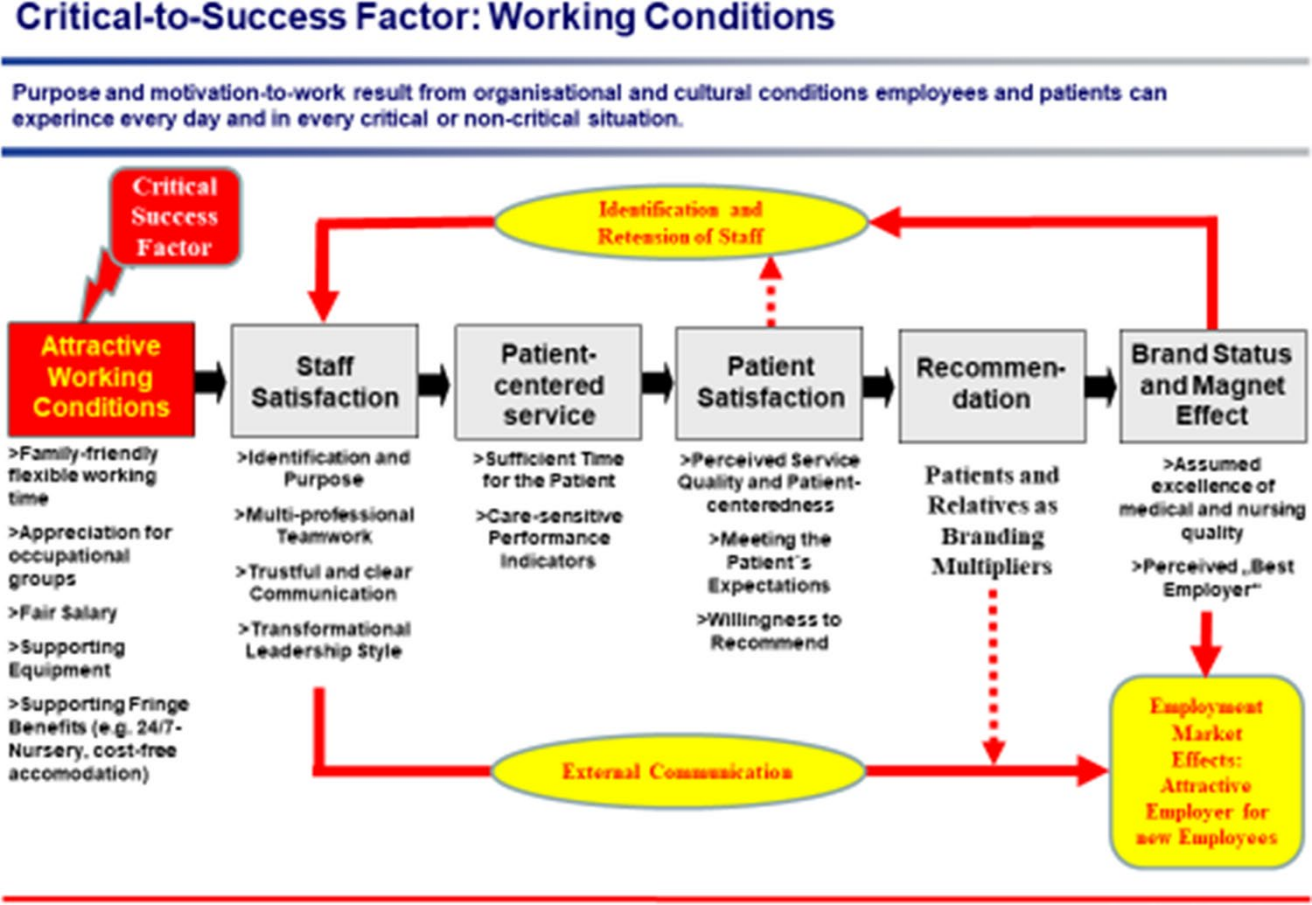
Attractive working conditions are the convincing instrument for staff retention and recruitment in the “war for talent” of hospitals ( Source: own representation)

Despite these well-known theoretical background knowledge occupational groups working at the patient’s bedside experience an identity crisis when working conditions become entrenched that give physicians and nurses a feeling of a lack of appreciation for their work, their commitment and their performance. Particularly the medical personnel in intensive care units, who are particularly challenged by the Corona pandemic, complain about a lack of appreciation (86%), about insufficient quality of personal protective equipment (48%), perceive a reduction in motivation (48%), point to additional burdens due to childcare (77%) and fear a deterioration of working conditions (93%) The current care ratio (nurse to patient per day shift) of 1: 2.7 is already well below the ratio of 1:1.2 recommended by the German Society for Intensive Care Medicine (DGIIN) (Karagiannidis et al. 2020).

During the Corona crisis the working situation especially for nurses working on an intensive care unit (ICU) has dramatically worsened. Only one Corona infected patient ventilated on an ICU requires the five-fold capacity of nursing resources compared to a normal acute care patient and a Corona patient’s length of stay (LOS) is four times higher than average. Hence, one Corona patient treated on the ICU causes that up to ten other acute care patients suffering from serious diseases like heart failures, cancer or cox-arthrosis cannot treated as instantly as necessary and were put on a waiting list.

Therefore, the working burden for the staff escalates to a permanent situation. As one consequence, 30% of the nursing staff were very outspoken about quitting their job as soon as possible. This may be the start of a doom loop leading to a dramatic gap in health care provision.

As a result of management failures in the past with regard to inadequate investment funding, restrictive staffing and equipment, and a failure to digitalize, employees in the medical care sector have been pushed to the limit by the Corona pandemic. In conjunction with working conditions that are experienced as dysfunctional, hostile to families and hazardous to health, and remuneration that is perceived as unfair and woefully inadequate, the question of appreciation arises for a growing number of employees, and with it the question of the meaning of performance and commitment, as well as career prospects in the context of activities that are not in direct contact with patients. This has massive consequences for the quality of medical care in the future.

## Findings from the Corona pandemic

The Corona pandemic has clearly brought three structural problem areas to the attention of the public as well as decision makers in politics, the medical industry and medical care providers:

1. The *vulnerability of global supply chains* (von Eiff 2020) for medical products (catheters, wound drains, kitpacks, custom procedure trays) and pharmaceuticals (propofol, oxytocin, epileptics) has caused a dangerous undersupply, especially in therapy-intensive areas (e.g., oncology). A lack of drug availability for tumor treatment has induced, among other things, a partial abandonment of first-line therapy (e.g., combined PEB therapy for testicular tumors) and required a change in patient treatment therapy mid-cycle. Supply disruptions of personal protective equipment have made medical care more difficult in terms of organization and timing and raised the risk of infection for medical staff and patients alike (von Eiff 2021).
  Corona has revealed that medical care quality depends increasingly on “low-cost routine products” as well. So, a tremendous shortage of personal protective equipment e.g. FFP2 masks, OR gowns and disinfecting agents (typical “bagatelle products” in non-COVID times) led to bottlenecks in patient care and hazardous situations for staff and patients. Another breathtaking example: the absence of sodium chloride irrigation for a transuterine resection leads to procedure cancellation. Thus, the absence of a product with a negligible cost in the single-digit euro range in turn causes a loss of revenue to the order of €7000 and impairs patient well-being (outcome). Such situations are frustrating for employees in the health sector, reduce motivation, and cast doubt on the meaningfulness of their work, and the esteem in which their profession is held, since, from their point of view, "false economies have been made”, i.e. in the wrong area.
2. The *digitalization gap*, i.e., the extremely low level of “digital maturity” of German medical service providers by international comparison, has proved to be an obstacle on the way to establishing a pandemic-compatible organization for effective infection management (Corona app disaster), as well as for platform-controlled supply logistics for system-critical products to match product availability and demand.
  The absence of telemedicine services, of platforms for virtual physician visits, of tele-monitoring options for patient care in the home, and of diagnosis- and therapy-supporting smart phone apps (von Eiff 2020) have led (due to fear of corona infection) to a sometimes alarming decline in patient numbers in medical practices OR doctors’ surgeries, hospitals, and emergency rooms (Mangiapane et al. 2021). The medical effects that emerge over time, and economic costs of a failure to screen for breast cancer screening (− 83%), skin cancer (− 70%), and diagnostic colonoscopies, as well as the consequences of the decline in outpatient treatment cases (− 23%), are not difficult to imagine.
3. The *shortage of specialists* in medical professions, which has built up over the years and is directly accompanied by a deterioration in working conditions, increases the risk of treatment errors and has a negative impact on the motivation and willingness to commit on the part of the professional groups working “at the bedside”. In the pandemic, it was not ventilators or intensive care beds but a lack of medical professionals, especially in nursing, that emerged as the crucial bottleneck in patient care.

In summary, it is safe to say that a multitude of sins in health care politics and hospital leadership over years became manifest and are now responsible for poor medical services and work overload for occupational groups working at the bedside.

## Causes of structural deficiencies: investment backlog, cost pressure and management deficits

Despite legal obligations, those with political responsibility have for years failed to provide investment financing for hospitals that would maintain their viability, and have additionally increased cost pressure on the medical sector through the DRG remuneration system. The resulting competition for service volumes has hindered the formation of centers, hampered the organization of health care networks, caused duplication of work between hospitals, wasted resources, and led to a loss of quality in medicine.

Hospitals have tried to counteract the cost pressure, which has been increasing for years, with two measures:

- Cutting costs by *reducing staff*, primarily in nursing, with the consequence of rising workloads and time pressure in patient care, deteriorating working conditions, increased hygiene risks, increased susceptibility of clinical processes to errors, thus jeopardizing the quality of care for patients, demotivated the dedicated staff and caused a flight into professions unrelated to medicine. At the same time, it became apparent that these stressful working conditions were not compatible with the life plans and professional expectations of Generations Y and Z entering the workforce (“work-life balance”). Added to this was what was perceived as inadequate pay in terms of performance requirements and responsibility. The urgently needed young talent was deterred and preferred a career path in other industries who were keen to employ them in the cross-industry “war for talent”.
- Cost cutting through the *procurement philosophy* of “price-oriented purchasing” led to the lowest price of a medical product driving supplier selection, rather than its effect on process efficiency and patient outcome, i.e. its value contribution. Short-term supply contracts in conjunction with low-price-oriented tendering procedures resulted in a “Lopez effect”: In his position as a chief purchasing officer of a big international automotive company Lopez leverages his purchasing power in order to put suppliers under severe price pressure. As a reaction, the suppliers reduced product quality to basic functionality in a cost-cutting manner. The identical reaction could be recognised in health care. If manufacturers of medical products were forced to low prices they react with cost cutting by value engineering to the disadvantage of product functionality, making the handling of medical products more cumbersome and worsening efficiency in clinical processes. The pressure exerted by purchasing managers and controllers to use cheap medical products, which are associated with cumbersome handling and risks of use, was perceived by nurses and physicians as expressing a lack of appreciation of their work.

This one-sided *economization of medicine* aimed at cost reduction has contributed to the fact that medical professions have begun to doubt the meaning and purpose of their work and have complained about the lack of appreciation.

The *medical industry* reacted to this price pressure with a value-analytical optimization of products (again, Lopez effect: reduction to basic functionality) and the establishment of international supply chains according to the "economies of scale" principle, i.e. the selection of suppliers and production sites was based on the criterion of lowest cost. The economically driven concentration on a few production sites, in conjunction with an uncontrolled extension of supply chains with "extended workbenches" in emerging countries, exacerbated their susceptibility to supply disruptions and reduced the security of supply for the health sector.

## Components of a “value-based leadership model”

### Leadership value system

Convincing and successful leadership conveys binding values and standards (*what is the purpose of our actions and what guides them and our thoughts*?) in a complex VUCA world (*volatile, uncertain, complex, ambiguous*) and creates legitimacy (*for whom do we create added value and what kind exactly?*), provides orientation (*what are we striving for and what are we doing to achieve this?*), and knows how to transform plans into successful corporate development (*how do we achieve it?*).

Leaders are assisted by the model of “value-based leadership in healthcare” (see appendix Fig. 2). It serves as an action orientation and compass for management in the health sector. In particular, it involves linking the cultural and behavioral aspects of an organization with organizational and decision-making structures that create transparency, control instruments, and proven leadership practices.

**Fig. 2:**
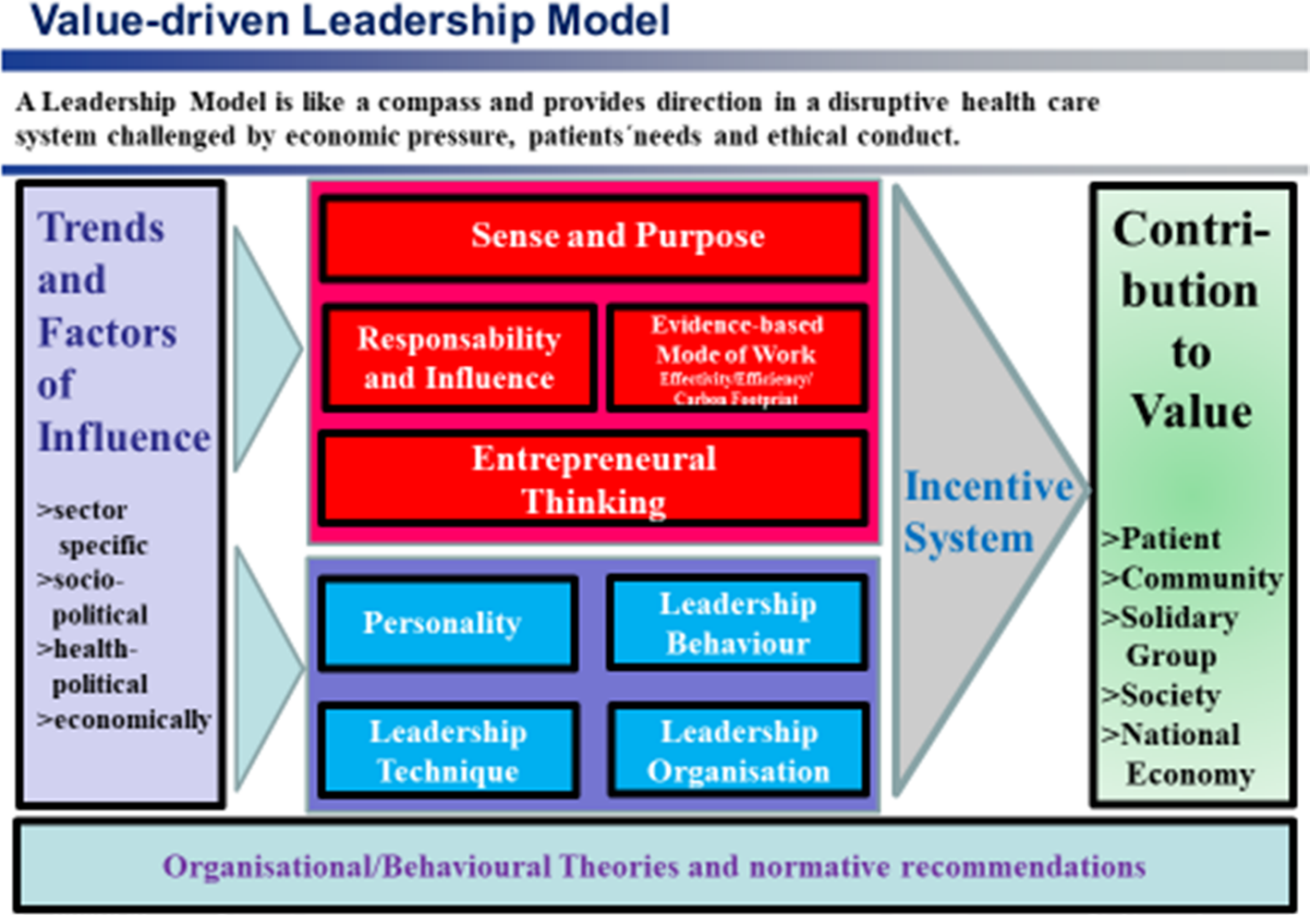
Structure and design dimensions of a “value-based leadership model for the healthcare sector” (Source: own representation)

Of particular importance are leadership values and leadership action guidelines, but also concrete instruments and leadership techniques that contribute to successful leadership.

Leadership values are represented by the design dimensions of meaning and purpose, responsibility, entrepreneurial thinking (“every employee is a problem solver”, Scott Myers 1981; Liker 2004) and resource orientation (= efficiency, effectiveness, sustainability, avoidance of waste). The questions to be answered are: “What mode of thinking characterizes leadership?”, “Which ethical rules guide leadership?” and “How are the efficiency and effectiveness of clinical processes ensured in order to achieve sustainable financing of the health sector?”.

Sense and purpose (“Purpose”) determine the legitimacy of a company in the competitive market and in the community. Sense and purpose show what the customer (patient, family member, referrer, cooperation and business partner, community) is entitled to expect from a company. Internally, sense and purpose are seen as the central source of intrinsic motivation for employees and, in the sense of an overarching (meta-) goal, as “arbitrators” of conflicting goals between economic and medical requirements. Externally, sense and purpose characterize the company's role in the provision of services of general interest in a solidarity-based healthcare system and its contribution to society.

### Leadership dimension “responsibility” in the health sector

The Corona pandemic has shown that the “*leadership dimension: responsibility”* has a unique significance in the health sector (see appendix Fig. 3). All decisions and actions, whether organizational, financial, procurement-related, or therapeutic, are primarily directed toward ensuring the well-being of the patient and are subject to the medical ethical maxims of “primum nihil nocere”, “patient well-being”, “autonomy”, and “dignity” (Wiesing 2020; von Eiff 2014).

**Fig. 3:**
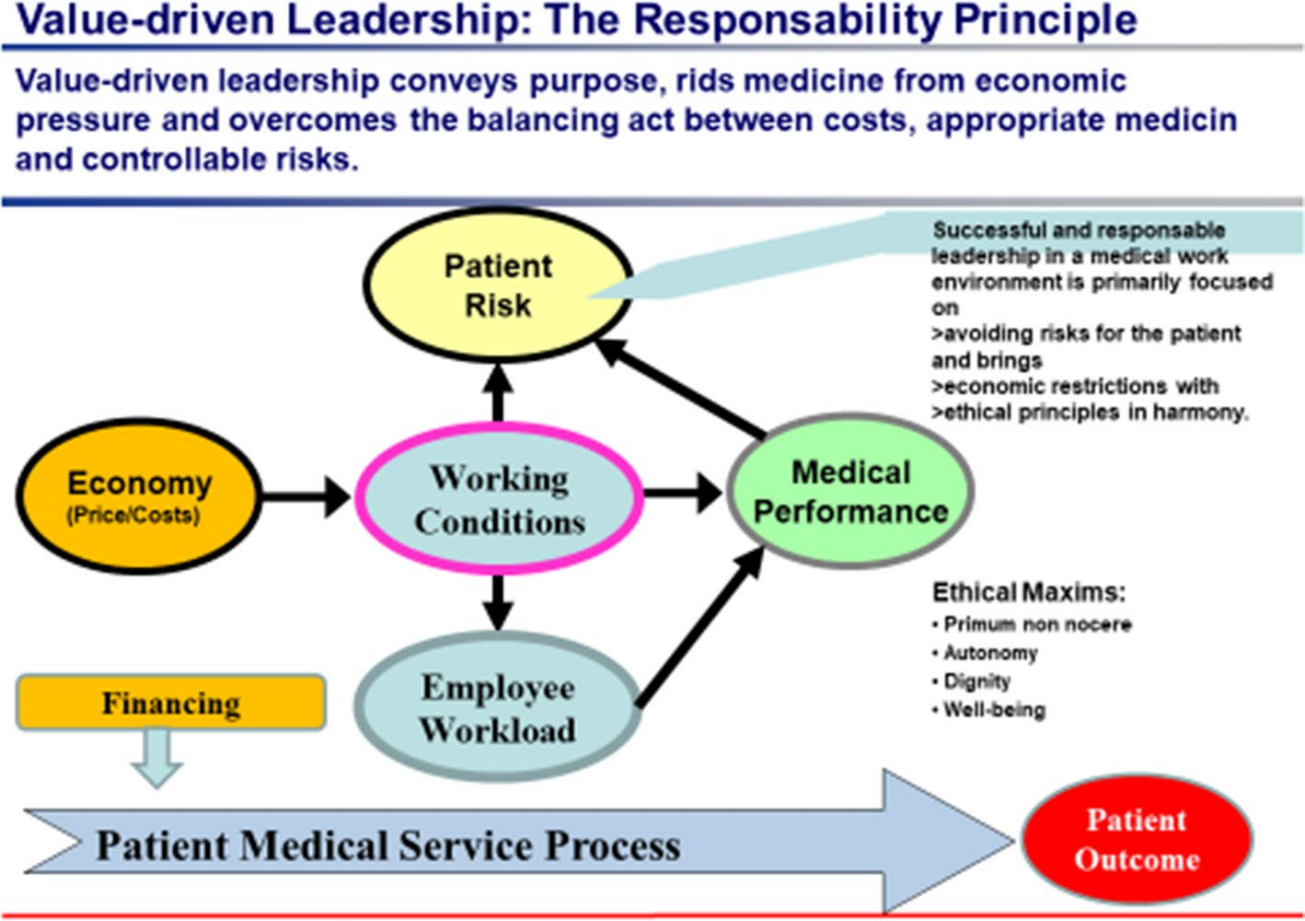
Value-based leadership considers the impact, in every decision, of investments, medical products and organizational changes on possible patient risks in accordance with the principle “above all, do no harm” (Source: own representation)

Economic principles (e.g., causality principle, principle of equality) are used in the spirit of this responsibility to address the challenges confronting the health care system in terms of sustainable financing and a fair allocation of health care services.

The assumption of responsibility is reflected in the ethical criterion of the extent of risk borne by the patient when, for reasons of cost or resource scarcity, rationalization is conducted in the health care sector, a cheap medical product is procured that requires more work and effort, or the necessary measures increasing patient safety are sacrificed (e.g. patient-centered drug safety according to the principle of “Closed Loop Medication Administration”; von Eiff 2021).

In that sense, leadership is not challenged achieving shareholder value, but is responsible contributing add-value to patients (securing medical and emotional needs), employees (providing motivating working conditions), community (economic and social engagement) and environment (carbon footprint reduction).

### Leadership dimension “contribution to value”

This dimension copes with the value managers provide to the stakeholders of a hospital (patients, relatives, general practitioners), to the society at large and to the community the hospital is located in.

Leaders are not only responsible for the economic success of the hospital, but have also to keep in mind the consequences of their decisions for society and environment.

Hospitals are an integrated part of the infrastructure of a region and as such contribute to citizens’ wellbeing, safety and quality of life. As employers hospitals could basically offer attractive, challenging and panic-prove jobs and additionally represent an important economic factor.

Bearing this in mind, leaders are on duty to focus on a value-based health care strategy (see appendix Fig. 4).

**Fig. 4:**
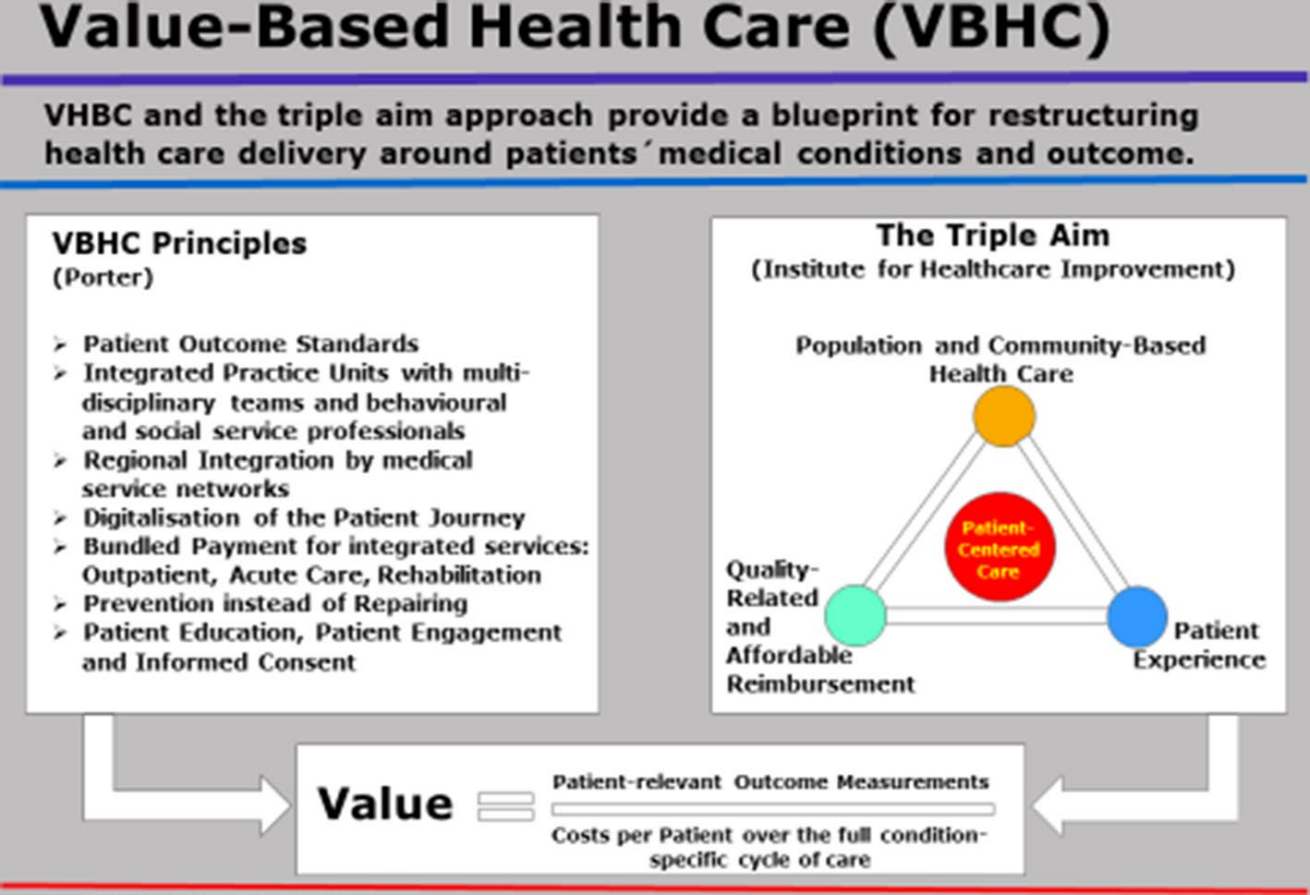
The principles of value-based health care and the strategic orientation of the triple aim approach (Source: own representation)

The Value-Based Health Care Approach (VBHC) contains of several principles (Porter/Teisberg 2006; Porter/Kaplan 2017):

Principle 1: Medical care is allocated in integrated practice units and performed by multi-disciplinary teams containing of various types of clinicians, supported by dedicated behavioral and social service professionals.

Principle 2: Measurement of outcomes and costs are operated at the level of the patient´s medical conditions. Patient outcome is measured by clinical and patient-reported outcomes (PROMS). The total costs of treatment are analyzed by using time-driven activity-based costing or the cost per patient over the full cycle of treatment (Life-Cycle Cost Approach; von Eiff 2020 a).

Principle 3: Reimbursement of medical services is oriented to “Bundled Payment”. This is an approach to pay for complete, integrated treatments along the patients´journey through the continuum of care.

Principle 4: Prevention through life-style nudging instead of putting the focus on “medical repairment”.

Principle 5: Cross-sectoral, structured co-ordination of care along the complete patient journey by gatekeepers and case managers.

Principle 6: Regional integration in cross-sectoral medical networks consisting of primary care physicians, specialized clinics and rehab facilities, so the full patient journey is covered (von Eiff 2016). On this basis, high quality expansion can be rolled out for specific populations.

Principle 7: Digitalization is the “booster technology” to bring medicine to the patient and to make sure also people in rural areas have access to medical services anytime. This is the key to achieving the basic target triangle in health care (quality, access, affordability).

From a strategic perspective VBHC is strongly related to the “Triple Aim Approach” (Bisognano/Kenny 2012) and the “Boundaryless Hospital Approach” (Albach et al. 2016).

The boundaryless hospital is patient-centered, provides a safe clinical environment, ensures coordinated care along the continuum of care in networks of specialized caregivers and incorporates the mission of transforming a typical health care setting into a healing environment that improves patient outcomes and employee motivation.

Triple aim contains of the three dimensions “community-based services” (including medical services, preventive life-style support, gatekeepers and case management coordination), “patient experience” and “quality-related reimbursement”.

The dimensions “population and community-based services” and “patient experience” represent the patient´s journey oriented to the medical and social needs of populations instead of the episodic approach so far common in medicine.

A population contains of human beings with some predefined criterion in common and tight together due to similar characteristics e.g. diabetes, cancer entities, ethnic or migration background. Hospitals are challenged not alone to offer acute care and rehab services, but also operate “prevention facilities”, which aim to nudge medical behavior towards a health promoting lifestyle. So, prevention facilities are expected to contribute to a better community health and a lower number of (unnecessary) hospital admissions.

The dimension “quality-related and affordable reimbursement” represents the incentive system set by politicians and payers in order to encourage hospitals performing medical services oriented to strongly defined quality measures (Donabedian 1988): outcome quality (mortality, morbidity, complications, satisfaction), structure quality (staff, equipment, guidelines, supplies), process quality (interaction, pain, procedures) and indication quality (precision and timeliness of diagnosis). The quality dimension “social quality” (von Eiff 2000) includes the quality indicators “employee satisfaction”, “work environment” and “working conditions” (Aiken et al. 2011).

VHBC (Porter) and Triple Aim Approach (IHI) are complementary concerning the perspective of qualified and affordable health maintenance services. While Triple Aim is focused on the inside and outside perspective of the patient journey, the Porter approach aims on providing a most effective and efficient organization of medical services: multidisciplinary teams inside the hospital, hospitals structured as specialized integrated practice units and regional integration by medical networks as outside perspective.

Both approaches recommend innovative modes of reimbursement (e.g. bundled payment, capitation, regional budgets).

### Reliability of leadership

Another experience from the Corona crisis with relevance to leadership is the importance of *reliability* in statements, *predictability* in behavior in combination with clear, *distinctive communication*.

The reliability and predictability of a leader refers to setting the rules of the game for dealing with each other, and the manner in which violations of these rules are sanctioned by management. “Walking the talk” or the “identity of talk and action” (von Eiff 2000) are essential for leadership effectiveness and acceptance.

At the beginning of the pandemic in March 2020, the message was, “Wearing protective mouth-nose masks has no pandemic protective effect and may even be associated with health risks”. A few weeks later, a variation of that message circulated: “Protective masks do not protect the wearer, but slow the spread of viruses when an infected person wears a mask”. The next communication variant: “If everyone wears a mask, of any kind, there is a protective effect for everyone”. And after that came the variant: “Only FFP-2 masks, worn according to regulations, provide largely reliable protection”. Finally, masks were made compulsory in public places. The communication surrounding daycare center openings and school closings was and remains downright bizarre. Here, the politicians’ claims that “children up to the age of twelve are not contagious or are less contagious than adults” and that “daycare centers and elementary schools do not pose a “risk of infection” were contradicted by the expert opinions of infectologists and epidemiologists. Through the scientifically untenable sham discussion about incidence values and reproduction rates, politicians failed to secure supply contracts with vaccine manufacturers at an early stage and to establish in good time, antigen testing concepts for pupils and teachers, vaccination priorities for educators, air filter equipment for classrooms and hybrid forms of teaching. With such constantly changing interpretations and opinions, leaders not only gamble away trust and acceptance, but provide an excuse for infectiologically alarming social behavior on the part of Corona deniers.

### Leadership in the crisis through team management

Corona has provided further insights with regard to established (centralistic, hierarchical) leadership concepts and practiced forms of cooperation—also with regard to their motivating effect or impact on the sense of meaning. “Crisis management at the bedside” is *team management*. The medical and social care of Covid-19 patients is complex, requires expertise from different disciplines and the interaction of different professional groups.

An effective treatment method was not available at the beginning of the pandemic and specific drugs simply did not exist. The treatment process was driven by trial and error: face-down positions, intubation, ECMO therapy, ventilatory pressure. Through the mutual exchange of experience and joint development of processes and their unbureaucratic implementation, learning processes took place in the team, with a high degree of individual autonomy and responsibility. The teams experienced defeats (deaths despite weeks of intensive treatment), but were also able to share successes. The development of team spirit and the possibility of “being able to make a cause one’s own”, “to achieve visible successes through one’s own initiative” (von Eiff/Stachel 2006) makes it possible to “find the work meaningful”. This important design approach of a value-oriented leadership model is reflected in "integrated therapeutic teams”, “heart teams”, “rapid response teams”, etc. as forward-looking forms of collaboration, as well as in innovative job descriptions such as “surgical technical assistant”, physician assistant”, “endo nurse”, and “stroke nurse”.

## Discussion and recommendations

### Corona and the limits of the economization of the health sector

Corona has also demonstrated that the economization of medicine is the wrong way to go. Services that cater to the interests of the general public cannot be achieved via competitive rivalry. “Shareholder value” is unsuitable as a business management principle in the health care sector, because of its short-term orientation and the dominance of financial ratios. The low-cost-oriented provision of medical staff and medical products leads to ethical dilemmas for doctors and nurses, as well as to doubts about their own medical and nursing mission.

The study on “Dealing with scarcity of funds and physician rationing” (Strech 2014) revealed that 68% of the surveyed physicians confirmed that they were no longer able to provide all useful services to state insured patients, due to limited funding.

In the study by Ghanem et al. (2015), 82% of physicians reported that increasing cost pressure was affecting their job satisfaction. In the KBV study, 68% of junior physicians complained about the “strong economic pressure” and time pressure when caring for patients (Hillienhoff/Osterloh 2019; Korzillius 2020).

A survey by the Professional Association of German Surgeons (Siewert et al. 2021) documents the consequences of the economization of medicine: ethical conflicts due to reduced lengths of stay, patient selection, omission of unprofitable medical care, and introduction of lucrative surgical methods. The pressure exerted by regular reporting of economically relevant performance figures conflicts with physicians’ values; professional satisfaction suffers as a result, and the associated conflicts of objectives exacerbate the shortage of junior physicians. Due to economization, the very point and purpose of certain medical services is increasingly called into question. Here, leadership is called upon to ensure that role conflicts between economic profit maximization and medical values are avoided.

### Action steps for successful leadership: the post-pandemic perspective

The post-pandemic catalog of measures for successful leadership should focus on fields of action that have an impact within a short timeframe and take into account the primary concerns of the professional groups working on patients. There is no time for holistic, organization-wide change processes in leadership forms, organizational structures, work processes and the corporate culture, and it is no longer possible to communicate such a conceptual understanding to employees.

#### Working conditions

The *working conditions* in the core professions of the medical business must be adapted to the requirements of employees and the conditions of the labor market. Working hours need to be designed in a family-friendly manner and oriented towards the findings of research on work processes, risk management and occupational health management. A program of measures also includes improving the pay situation, support with fringe benefits (e.g. free daycare places with 24/7 care, housing subsidies in densely populated and expensive areas, reimbursement of travel costs) and the introduction of innovative working time arrangements.

The prerequisite is *forward-looking workforce planning* in conjunction with a clear strategy for the recruitment, selection, induction and development of new employees. Above-average turnover rates, an increase in burnout syndrome, short retention periods in the profession, aging among nurses and physicians, migration from medical professions to administrative professions, are all clear signs of long-standing structural failures in personnel policy.

If the number of overtime hours in nursing is rising and the number of night shifts per physician increasing, and at the same time vacant positions are deliberately not filled for months in order to save costs, this is also the result of a personnel policy that operates according to the maxim: “Cost reduction before employee protection and patient safety”.

All these measures presuppose a management that is professionally competent and willing to provide sustained support to employees, and whose personnel policy objective is to create an “*attractive workplace*” (see appendix Fig. 5). Attractive working conditions are *the* success factor in the “war for talent” of hospitals. They enable patient-centered work as well as employee satisfaction, and lead to a “magnet” status via patient satisfaction and willingness to recommend the hospital to others. Leadership is the success factor of necessary change in the “working world: hospital”. Leadership shapes the organizational structure and corporate culture by actively setting an example and creating a conducive environment. The culture of collaboration and the values according to which people think and act are decisive for the agility of an organization, for its ability to innovate, and for its safety with regard to patient risks.

**Fig. 5:**
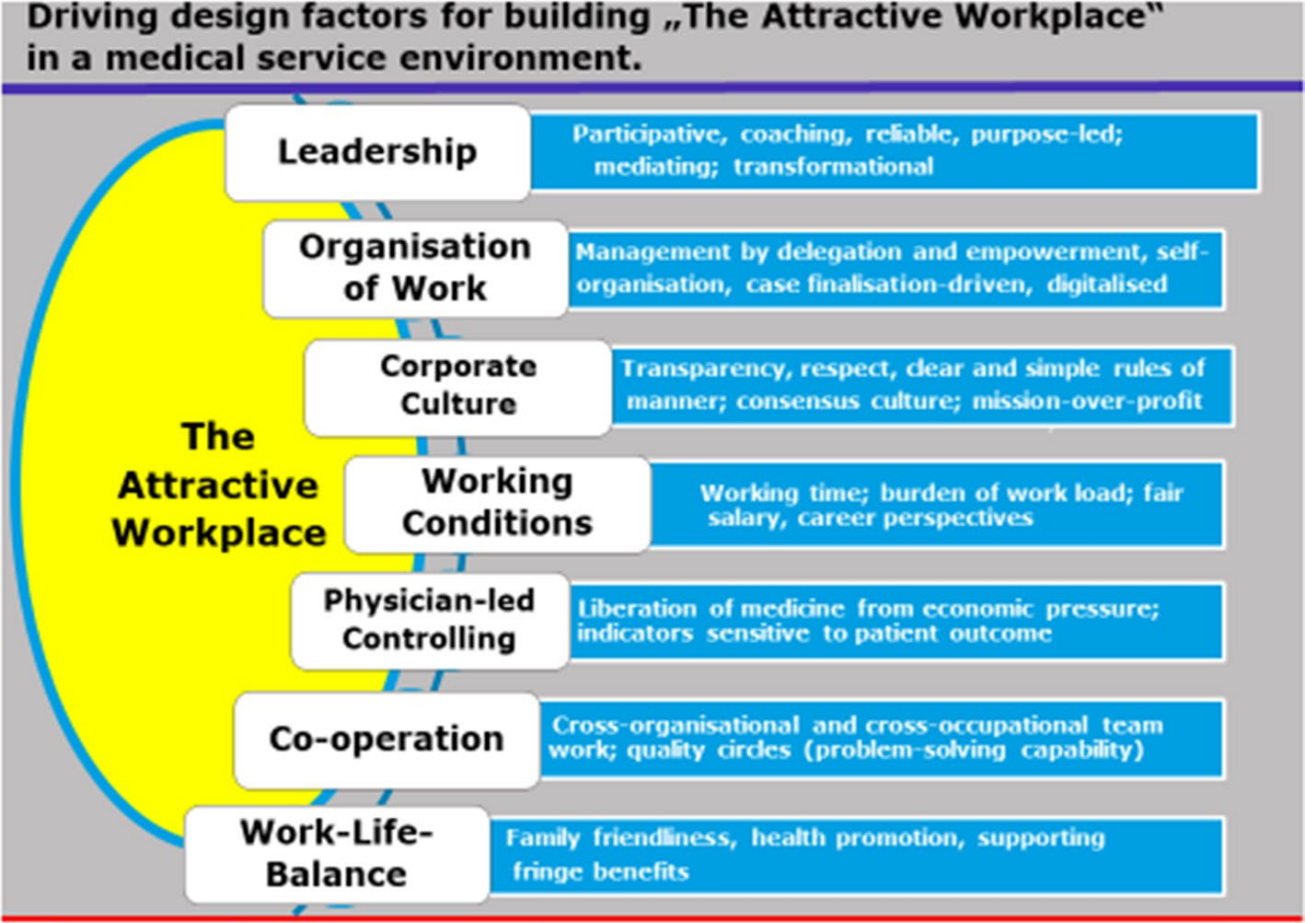
The design elements for the “attractive workplace” in the medical establishment are derived from employee complaints about the fundamental situation and from the demands placed on the working environment by generations Y, Z and C, as well as the feminization of medicine (Source: own representation)

Management is called upon to implement the abovementioned individual measures to improve the attractiveness of the “hospital as a place of work”, on the basis of a manage ment philosophy that focuses on patient well-being, patient safety and employee satisfaction and is thus “automatically” also economically successful. The example of the MAYO Clinic, which is known for its international excellence status (“The Clinic”), shows that this can work.

MAYO stands for excellent medicine and patient-centered care, is known for a management culture of mutual respect among professional groups, a clear medically oriented value system (“most of all, do no harm”) and a “relativized” importance of economics in the health sector.

The MAYO leadership philosophy is “physician led”: every decision, every action, whether it is about the procurement of medical products, the provision of medical technology equipment, the service/offer for patients or new digitally based organizational forms of patient care, is put to the test of physician standards of medical ethics. Money and financial stability are important decision parameters, but money (or low cost) does not effectively prioritize or determine any decision: “Money is important, but money doesn´t drive the bus!” (Berry/Seltman 2008).

Controllers, financial officers and human resource managers, as well as administrators, are partners in the decision and service process, but they are not equal partners, and are limited to their support role: “Administrators are partners, but they are not equal partners, and this is purposeful” (Berry/Seltman 2008). And: All processes are oriented towards the patient (“patient first”: patient-centered care, Berry/Bendapudi 2003).

#### Digitalization of health care: closing the gap

No other area in healthcare has as much potential for increasing process efficiency, improving medical quality as well as patient safety, and ultimately achieving sustainable economic success, as the area of digitalization.

In particular, applications in the areas of quality assurance, drug safety, knowledge management, OP management, supply chain management and precision medicine, all contribute to process efficiency.

The goals of digitalization are challenging, and include improving patient outcome, patient safety and medical quality, while simultaneously reducing costs by cutting red tape and optimizing processes. Furthermore, a noticeable contribution to eliminating the shortage of skilled workers is expected.

Successful digitalization requires a fundamental reorganization of work, information and decision-making processes in the medical sector. New forms of care, from telemedical diagnosis via smartphone to digital ‘visits’, patient monitoring at home, patient-specific production of hip implants with 3D printers, and gene sequencing for early diagnosis of serious diseases, will become possible over the course of digitalization.

Digital treatment platforms with virtual physician consultations (see appendix Fig. 6) reduce the number of contacts in a pandemic situation, simplify documentation and billing processes, enable parallel second opinion calls, make more efficient use of scarce physician resources, and increase and broaden the availability and geographic coverage of medical services in urban and rural areas as well.

**Fig. 6:**
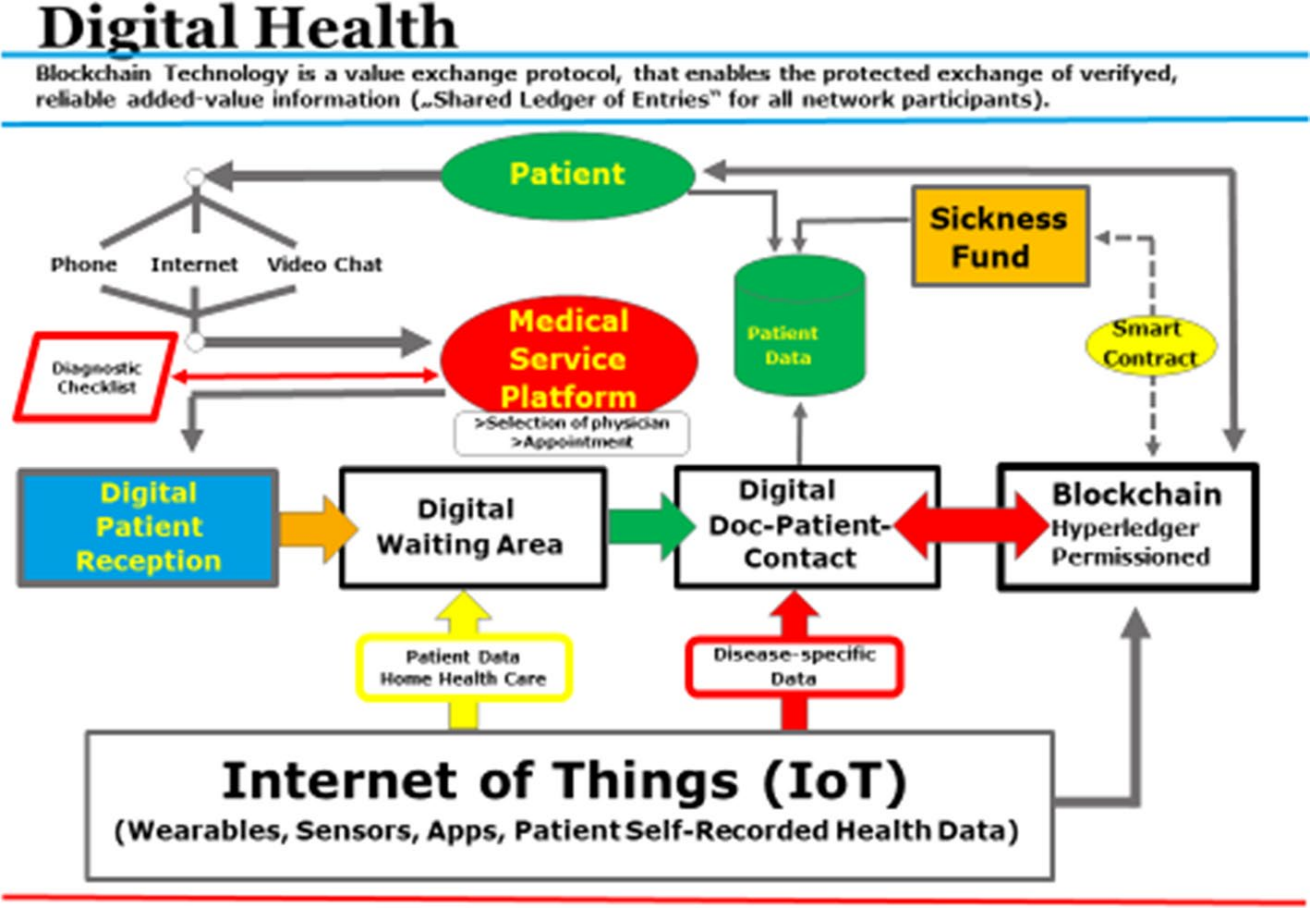
Digitalization of the medical sector is fundamentally changing care processes, the doctor-patient relationship and forms of collaboration: leadership is required from change managers (Source: own representation)

All this goes hand in hand with massive changes in the organization of clinical processes, forms of collaboration, and communication requirements. The doctor-patient relationship will also change fundamentally; medicine will come to the patient, and treatment decisions will be made according to the principle of “informed consent”.

Against this background, digitalization is not only a strategic and operational management task, but also a challenge for employees in particular. Here, leadership is called upon to steer the process of digitalization through professional change management.

#### Value-based procurement management

Perhaps the most important insight of the Corona crisis for the future strategic reorientation of procurement management is that the hospitals guided by a “cost cutting” and “cut-price-driven” procurement philosophy were affected first by supply disruptions, and existing customers were supplied preferentially. Supplier relationships are economically successful and resilient in the event of a crisis, only if they are based on trust, fairness, reliability and commitment. Strategic partnerships with the contractual components of risk sharing, process integration, change management and project management support, investment interlocking and innovative financing, will therefore shape procurement networks in the future.

*Value-based procurement management* is not based primarily on the price of a medical product or its cost. Every purchasing decision is based on value criteria such as patient safety, process efficiency, workload reduction and patient outcome. In addition, the public- good aspects of a “fair supply chain” (fair remuneration, prohibition of child labor, occupational health and safety) and “sustainability” (use of “cradle-to-cradle products”) are taken into account, as is the supply security of a supply chain (see appendix Fig. 7). These value criteria are compared with the life cycle costs of a product.

**Fig. 7:**
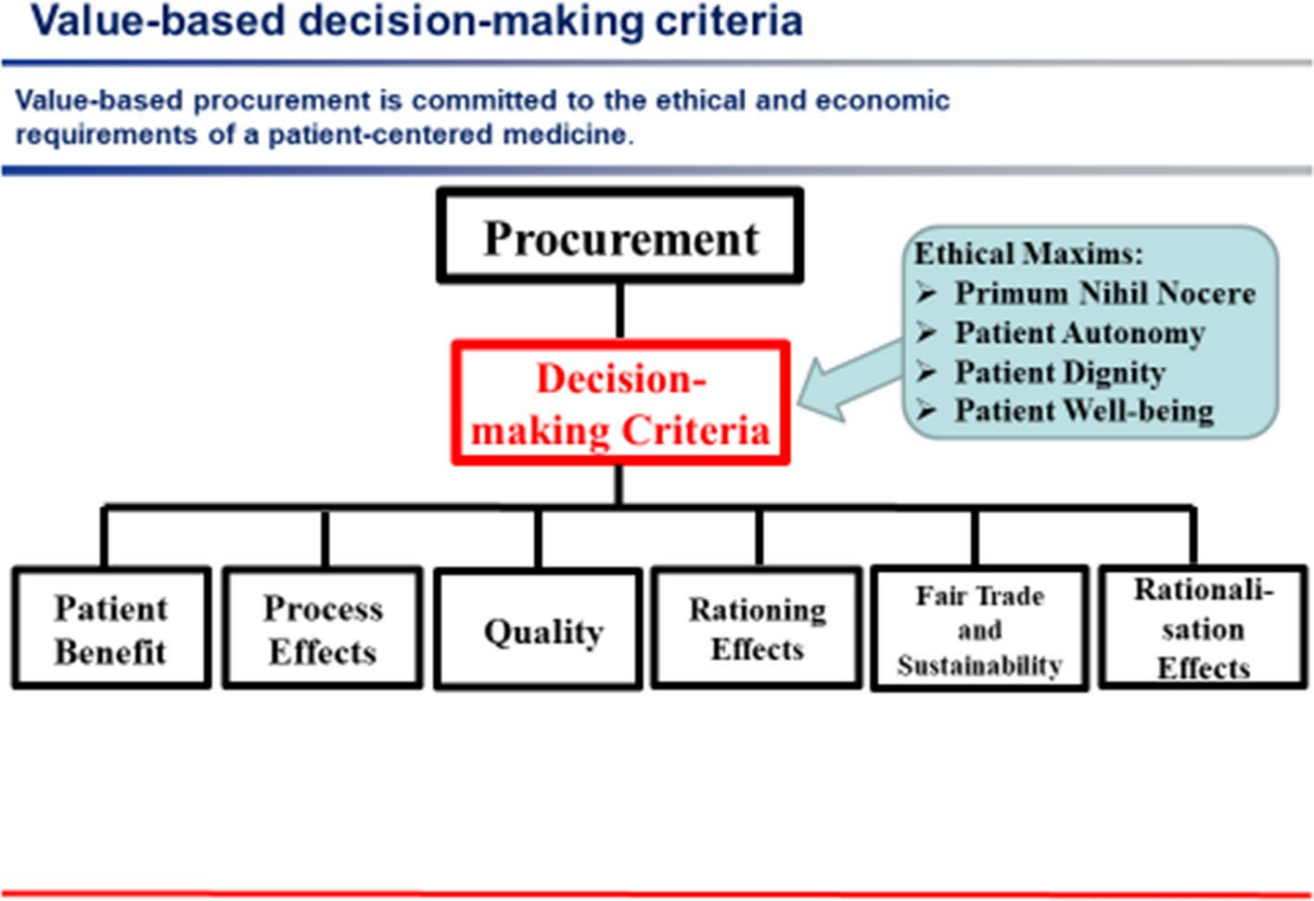
Value-based procurement management is oriented toward the benefits for users and patients, as well as to the common good criteria of sustainability and fair supply chains (Source: own representation)

## Conclusion

In the current labor market and workplace situation in hospitals, it is not a matter of adapting the supposed "best" leadership concept, but of finding one's own way to develop one’s own leadership culture that:

- has a clear vision and conveys a sense of purpose of the work,
- cultivates a collaborative culture of transparency and mutual respect,
- is patient-centered,
- provides smooth digitally-based workflows and supportive medical equipment,
- professionalizes employees’ “hands-on” skills and increases their stress-resilience through coaching, mentoring, and supervision,
- creates family-friendly and health-promoting working conditions, and
- establishes a fair compensation and incentive system that includes the vital components of individual compensation, team compensation and fringe benefits.

The form of “team management” induced by Corona requires astute management to ensure forms of organization and cooperation that grant individuals autonomy of action and decision-making, while at the same time enabling goal-oriented cooperation between professional groups as well as specialist functions. This type of leadership does not control hierarchically, but relies on entrepreneurial awareness at the employee level. Management tools include the creation of a shared problem-solving mindset, feedback-oriented communication, coaching, mentoring and supervision, and the orientation of actions toward outcome indicators that are sensitive to the nature of nursing care.

Working conditions and pay are currently the critical success factors for leadership and human resource policy when it comes to winning the “war for talent” as a hospital. The goal of human resources policy must be to design an “attractive workplace within the medical enterprise”.

## Appendix

See Figs. 1, 2, 3, 4, 5, 6, 7.
